# Neurenteric Cyst of the Posterior Cranial Fossa: A Case Report and Literature Review

**DOI:** 10.7759/cureus.22628

**Published:** 2022-02-26

**Authors:** Marcos V Sangrador-Deitos, Tamara E Sánchez-Cantú, Juan P Navarro-Garcia de Llano, Luis A Rodríguez-Hernández, Víctor Alcocer-Barradas

**Affiliations:** 1 Neurosurgery, Instituto Nacional de Neurología y Neurocirugía Manuel Velasco Suárez, Mexico City, MEX; 2 Diagnostic and Therapeutic Radiology, Hospital Español de México, Mexico City, MEX

**Keywords:** posterior fossa tumor, cystic lesion, posterior fossa, neuroenteric cyst, neurenteric cyst

## Abstract

Neurenteric cysts (NCs) are rare benign endodermal lesions of the central nervous system (CNS), most commonly found in the spinal cord. Intracranial lesions are rare, among which the posterior fossa appears to be the predominant location.

We present a case of a 60-year-old man who presented with a suddenly decreased level of consciousness. After a series of radiological studies were done, a multilobulated cystic lesion in the right posterior fossa was observed. Surgical resection was performed and based on its histopathological characteristics, NC diagnosis was confirmed.

Because of the wide list of differential diagnoses and low specificity of radiological features, surgical gross total resection remains the most effective treatment, followed by diagnosis confirmation through histopathological techniques.

## Introduction

Neurenteric cysts (NCs) are rare benign endodermal lesions of the central nervous system (CNS) defined as "cysts lined by mucin-secreting epithelium resembling that of the gastrointestinal tract," according to the 1979 World Health Organization (WHO) Classification of Tumours of the Central Nervous System [1]. In the 1993 classification, NCs were classified in the "cysts and tumor-like lesions" category; however, since the third edition was published in 2000, this category was deleted, and NCs are no longer considered CNS tumors. Previously known as enterogenous, enterogenic, enteric, and gastroenteric cysts, these lesions account for 0.01% of CNS tumors, three times more common in the spine than in the brain, accounting for 0.3-0.5% of spinal tumors [2-6]. Due to their uncertain pathogenesis, several hypotheses have been proposed. The most common theory suggests that during the third and fourth weeks of embryogenesis, the neurenteric canal's involution fails, which communicates the notochord (ectoderm) with the enteric tube (endoderm), so when endodermal cells abnormally separate from the gut, an NC appears [7]. In this article, we present a literature review and include a representative case.

## Case presentation

**Figure 1:**
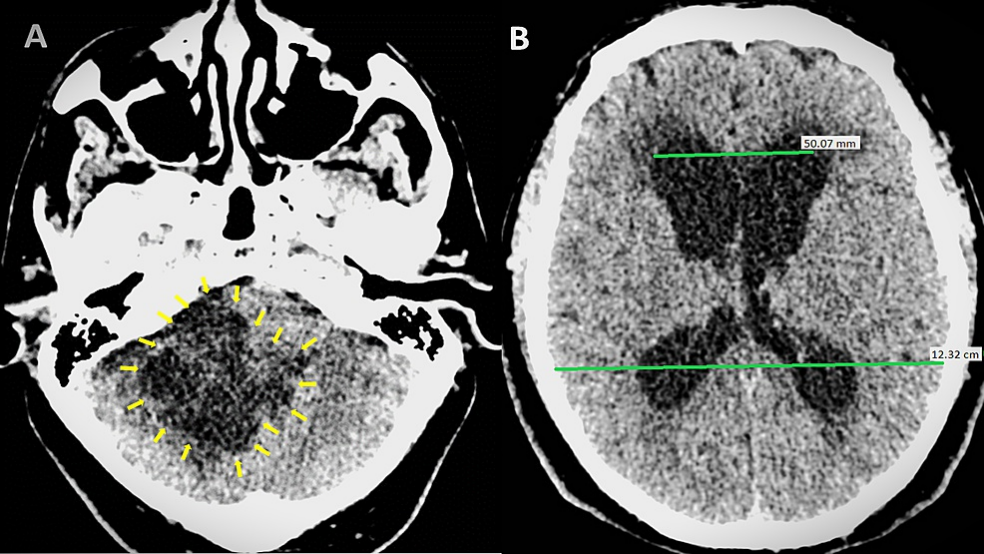
Non-contrast CT scan. (A) Axial scan at the level of the posterior fossa showing a multilobulated hypodense mass (yellow arrows). (B) A supratentorial compartment in which acute hydrocephalus was observed with an Evans index of 0.4 and transependymal edema.

A 60-year-old man with a history of intermittent headaches presented to the emergency room due to a suddenly decreased level of consciousness, with a Glasgow Coma Scale (GCS) score of 12 (M = 5, V = 4, E = 3). A computed tomography (CT) scan revealed a right posterior fossa hypodense mass with complete fourth ventricle obliteration, causing acute hydrocephalus (Evans' index of 0.4) [8] and brainstem compression (Figures 1A, 1B).

The patient was admitted to the operation room, where a ventriculoperitoneal shunt was installed uneventfully. Complete recovery of mental functions was observed after shunt placement.

**Figure 2:**
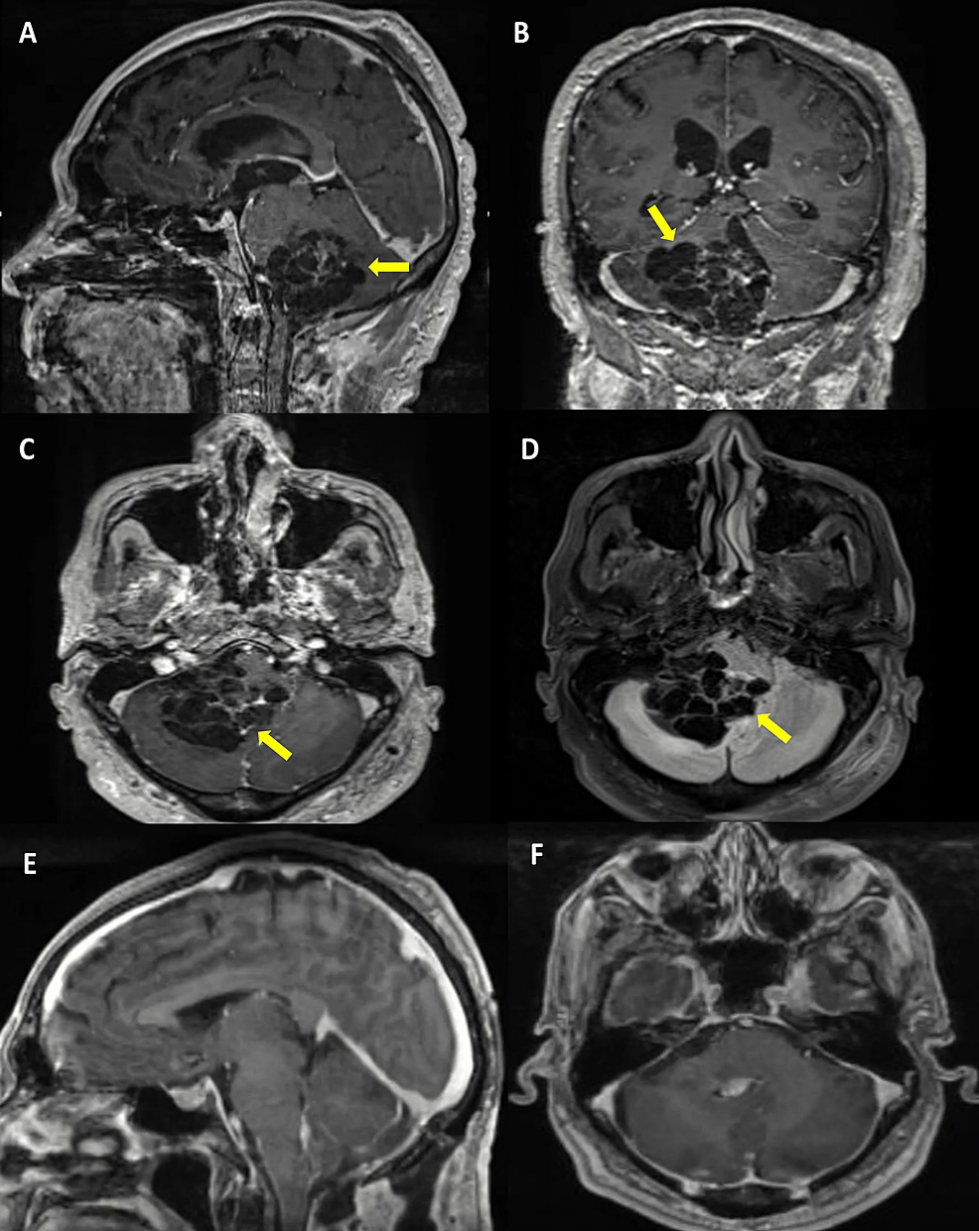
MRI images. (A) Sagittal, (B) coronal, and (C) axial postcontrast T1-weighted images demonstrated an intra-axial multilobulated hypointense mass posterior to the pontomedullary junction that does not enhance and appears to be extending into the fourth ventricle causing expansion on the rostral portion of this compartment (yellow arrows). (D) Fluid-attenuated inversion recovery (FLAIR) sequence in the same patient showed that the cyst fluid is hypointense (yellow arrow). No evident solid component was observed. Postoperative (E) sagittal and (F) axial T1-weighted with contrast images showing gross total resection of the mass.

A complete neurological examination revealed normal mental status, cranial nerve function, strength, and sensation, followed by magnetic resonance imaging (MRI), which in the T1-weighted sequence showed an intra-axial, multilobulated, hypointense mass dorsal to the right pontomedullary junction. This mass did not enhance after gadolinium administration and appeared to be extending into the fourth ventricle, causing partial expansion of this region. Contrary to the typical hyperintense descriptions, the cyst was hypointense on the fluid-attenuated inversion recovery (FLAIR) sequence (Figures 2A-2D). With all these findings, a preoperative diagnosis of hemangioblastoma was thought. Five days after admission, we performed a middle suboccipital approach, coursing through the telovelar corridor to reach a heterogeneous mass formed by solid and cystic multilobulated components, in which yellowish watery fluid was aspirated. Despite its strong adhesions to the lower pons and upper medulla, complete resection was achieved. The postoperative course went uneventful, and a three-month follow-up MRI showed no residual lesion (Figures 2E, 2F).

**Figure 3:**
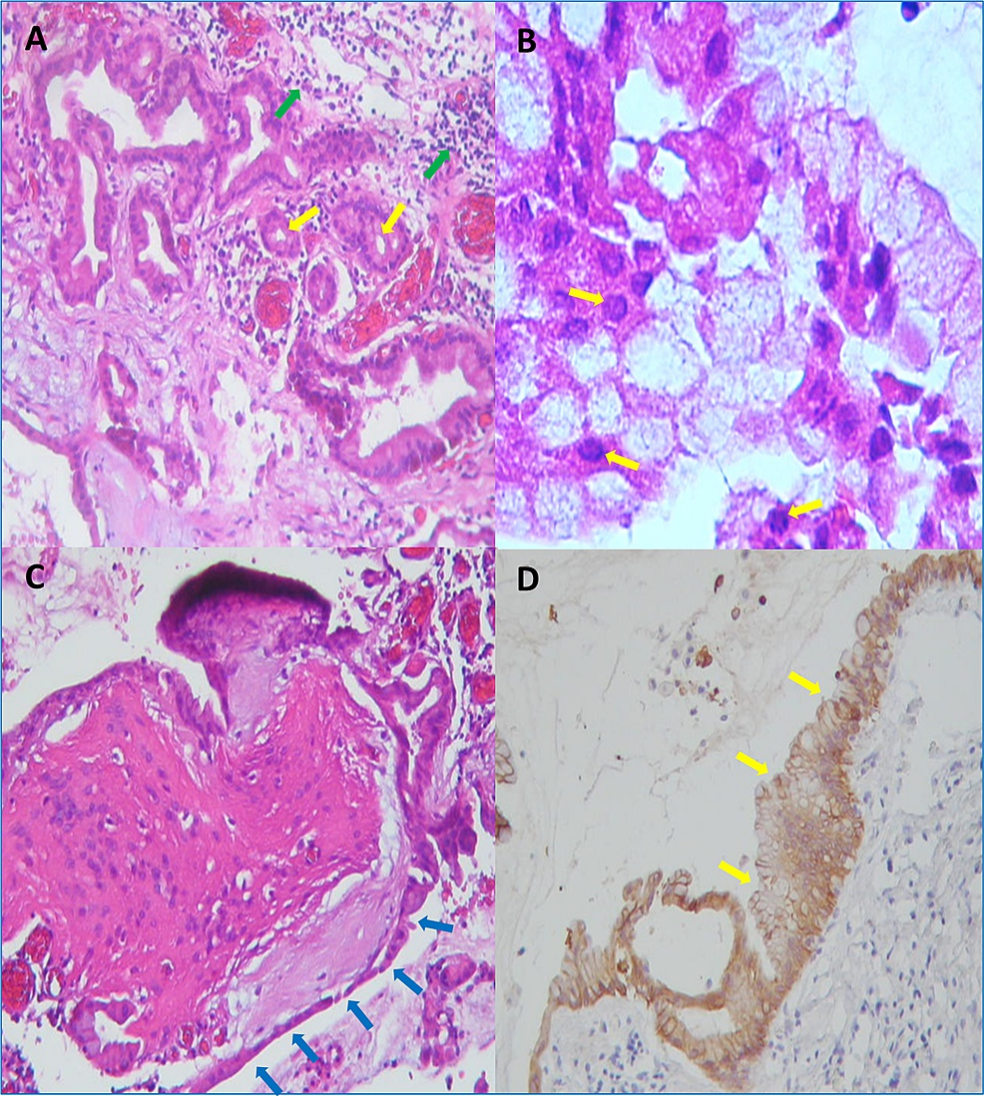
Histopathological findings. (A) The cyst wall was lined with a single layer or pseudostratified columnar and ciliated epithelial cells with mucin-producing goblet cells (yellow arrows), forming cystic and glandular structures accompanied by intense inflammation (green arrows pointing inflammatory cells) (hematoxylin and eosin, x10). (B) Goblet cells with strong periodic acid-Schiff (PAS)-positive staining (yellow arrows) (x40). (C) PAS staining shows intense affinity in the basal membranes of the epithelium (blue arrows) and the mucinous material (x40). (D) Positive immunostaining for cytokeratin 7 (yellow arrows) (x40).

Histological examination of the surgical specimen was compatible with the diagnosis of an NC, which, by light microscopy, resembled the gastrointestinal tract appearance with a single layer of pseudostratified cuboidal or columnar epithelium and intermingled goblet cells, exhibiting as well positive periodic acid-Schiff (PAS) staining. Immunostaining reported NC findings, such as cytokeratin 7 (ck7) and epithelial membrane antigen (EMA) positivity, and glial fibrillary acid protein (GFAP) negativity (Figures 3A-3D) [9,10].

## Discussion

Intracranial NCs represent 10-17.9% of all NCs and occur more often in the adult population. On the other hand, intraspinal NCs represent the most common location, which is more common in pediatrics [11]. Due to their rarity, the true incidence remains unknown. Posterior fossa lesions represent about 70-90% of all intracranial NCs, the majority located in the midline, with 34% extending into the cervicomedullary junction. According to the literature, only 14% are supratentorial [12]. The most common clinical presentation depends on the local mass effect. Posterior fossa lesions most frequently present with vertigo, hearing loss, tinnitus, and cranial nerve deficits, especially when located in the cerebellopontine angle (CPA) [13]. The slow-growing nature of these lesions often causes recurrent symptomatology over several years.

In 1976, Wilkins et al. proposed the first classification, which is still in use, based on histological features [14]. As intracranial NCs are rare, this classification is mainly used for intraspinal forms; however, it can be extrapolated to all NCs. Type A cysts have an epithelial lining composed of pseudostratified cuboidal or columnar cells, which mimic respiratory or gastrointestinal epithelium. Type B cysts are arranged in complex invaginations and have associated glands producing mucinous fluid. Type C cysts are associated with glial elements.

The imaging characteristics of NCs are variable. CT scan reveals a hypodense lesion with no contrast enhancement in most cases. Occasionally, iso and hyperdense lesions are observed, supposing a diagnostic challenge [15]. Thus, MRI remains the gold standard for evaluating NCs. Signal intensity may vary according to the protein content of the cyst. Hayashi et al. evaluated the biochemical contents of Rathke's cleft cysts, and the results proved that with protein concentrations of 10,000 mg/dL or less, signal intensity was low in T1-weighted images (WI) and high in T2-WI; with concentrations between 10,000 to 17,000 mg/dL, signal intensity was increased in both sequences; and with concentrations of 17,000 mg/dL or more, signal intensity was high on T1-WI and low in T2-WI [16]. However, the classical MRI findings are as follows: isointense in T1-WI, hyperintense in T2-WI, hyperintense on FLAIR sequences, and usually with a mild restriction on the diffusion-weighted image (DWI). As in CT, enhancement is rare. Differential diagnoses are arachnoid cysts, epidermoid or dermoid cysts, colloid cysts, Rathke's cleft cysts, parasitic, and cystic tumors [17]. There are no specific imaging features to make a consistent preoperative diagnosis.

Surgical resection remains the mainstay of treatment in symptomatic patients. An adequate surgical exposure to achieve complete visualization of the interface between the tumor and the brainstem becomes the primary objective during surgery. For lesions located ventral to the brainstem, a far-lateral transcondylar approach has been used, with good postoperative outcomes [11,18]. We performed a midline suboccipital approach using an interhemispheric telovelar corridor to reach the mass and achieve a gross total resection. In lesions located in the CPA, a retrosigmoid approach could also be an option. Gross total resection must always be pursued. Wang et al. described the relationship between the degree of adhesion of the lesion to the brainstem and the size of the lesion and the thickness of the wall, stating, "the greater the lesion's size, the thinner the wall of the lesion, the stronger the adhesion, and the more difficult the lesion is to be totally resected" [11]. Recurrence has been reported from four months to 14 years, even when total macroscopic resection seems to be achieved [19]. Cyst aspiration, fenestration, or partial resection are discouraged, as recurrence tends to occur sooner in these cases [20]. Thus, the surgical technique must be emphasized to achieve a total resection and reduce the risk of recurrence.

## Conclusions

Intracranial NCs are rare benign lesions that typically occur in young adults and should always be considered a differential diagnosis of cystic, intra-axial lesions of the CNS. An accurate preoperative diagnosis might not always be possible, as radiological features are not specific. When feasible, gross total resection is the most effective treatment method. Pathological examination and immunostaining must be performed to reach a correct diagnosis. Follow-up with MRI is advised for at least 10 years.

## References

[1] Zülch KJ (1979). Histological Typing of Tumours of the Central Nervous System. World Health Organization.

[2] Scoville WB, Manlapaz JS, Otis RD, Cabieses F (1963). Intraspinal enterogenous cyst. J Neurosurg.

[3] Small JM (1962). Preaxial enterogenous cysts. J Neurol Neurosurg Psychiatry.

[4] Aoki S, Machida T, Sasaki Y, Yoshikawa K, Iio M, Sasaki T, Takakura K (1987). Enterogenous cyst of cervical spine: clinical and radiological aspects (including CT and MRI). Neuroradiology.

[5] Veeneklaas GMH (1952). Pathogenesis of intrathoracic gastrogenic cysts. AMA Am J Dis Child.

[6] Gauden AJ, Khurana VG, Tsui AE, Kaye AH (2012). Intracranial neuroenteric cysts: a concise review including an illustrative patient. J Clin Neurosci.

[7] Christov C, Chrétien F, Brugieres P, Djindjian M (2004). Giant supratentorial enterogenous cyst: report of a case, literature review, and discussion of pathogenesis. Neurosurgery.

[8] Brix MK, Westman E, Simmons A (2017). The Evans' index revisited: new cut-off levels for use in radiological assessment of ventricular enlargement in the elderly. Eur J Radiol.

[9] Scaravilli F, Lidov H, Spalton DJ, Symon L (1992). Neuroenteric cyst of the optic nerve: case report with immunohistochemical study. J Neurol Neurosurg Psychiatry.

[10] Chen CT, Lai HY, Jung SM, Lee CY, Wu CT, Lee ST (2016). Neurenteric cyst or neuroendodermal cyst? Immunohistochemical study and pathogenesis. World Neurosurg.

[11] Wang L, Zhang J, Wu Z, Jia G, Zhang L, Hao S, Geng S (2011). Diagnosis and management of adult intracranial neurenteric cysts. Neurosurgery.

[12] de Oliveira RS, Cinalli G, Roujeau T, Sainte-Rose C, Pierre-Kahn A, Zerah M (2005). Neurenteric cysts in children: 16 consecutive cases and review of the literature. J Neurosurg.

[13] Bejjani GK, Wright DC, Schessel D, Sekhar LN (1998). Endodermal cysts of the posterior fossa. Report of three cases and review of the literature. J Neurosurg.

[14] Bruyn GW, Vinken PJ (1976). Handbook of Clinical Neurology: Tumors of the Spine and Spinal Cord. Handbook of Clinical Neurology: Tumors of the Spine and Spinal Cord.

[15] Kapoor V, Johnson DR, Fukui MB, Rothfus WE, Jho HD (2002). Neuroradiologic-pathologic correlation in a neurenteric cyst of the clivus. AJNR Am J Neuroradiol.

[16] Hayashi Y, Tachibana O, Muramatsu N (1999). Rathke cleft cyst: MR and biomedical analysis of cyst content. J Comput Assist Tomogr.

[17] Preece MT, Osborn AG, Chin SS, Smirniotopoulos JG (2006). Intracranial neurenteric cysts: imaging and pathology spectrum. AJNR Am J Neuroradiol.

[18] Liu JK, Couldwell WT (2005). Far-lateral transcondylar approach: surgical technique and its application in neurenteric cysts of the cervicomedullary junction. Report of two cases. Neurosurg Focus.

[19] Chaynes P, Bousquet P, Sol JC, Delisle MB, Richaud J, Lagarrigue J (1998). Recurrent intracranial neurenteric cysts. Acta Neurochir (Wien).

[20] Leventer DB, Merriam JC, Defendini R, Behrens MM, Housepian EM, LeQuerica S, Blitzer A (1994). Enterogenous cyst of the orbital apex and superior orbital fissure. Ophthalmology.

